# Anti-SMN complex antibodies in paediatric mixed connective tissue disease with interstitial lung disease: clinical and immunological insights

**DOI:** 10.1093/rap/rkag073

**Published:** 2026-07-02

**Authors:** Ann Tamura, Motoshi Sonoda, Sungyeon Park, Kota Nagasawa, Nobutaka Harada, Keishiro Kinoshita, Katsuhide Eguchi, Yasunari Sakai, Masataka Ishimura

**Affiliations:** Department of Pediatrics, Graduate School of Medical Sciences, Kyushu University, Fukuoka, Japan; Department of Hematology and Immunology, Fukuoka Children’s Hospital, Fukuoka, Japan; Department of Pediatrics, Graduate School of Medical Sciences, Kyushu University, Fukuoka, Japan; Department of Hematology and Immunology, Fukuoka Children’s Hospital, Fukuoka, Japan; Department of Pediatrics, Graduate School of Medical Sciences, Kyushu University, Fukuoka, Japan; Department of Pediatrics, Graduate School of Medical Sciences, Kyushu University, Fukuoka, Japan; Department of Hematology and Immunology, Fukuoka Children’s Hospital, Fukuoka, Japan; Department of Pediatrics, Graduate School of Medical Sciences, Kyushu University, Fukuoka, Japan; Department of Pediatrics, Graduate School of Medical Sciences, Kyushu University, Fukuoka, Japan; Department of Pediatrics, Graduate School of Medical Sciences, Kyushu University, Fukuoka, Japan; Department of Pediatrics, Graduate School of Medical Sciences, Kyushu University, Fukuoka, Japan

Key message

- Anti-SMN complex antibodies suggest a distinct subset of paediatric MCTD with interstitial lung disease.

Dear Editor, MCTD is an autoimmune disorder characterized by overlapping features of SLE, SSc and PM, together with anti-U1-RNP antibodies [1]. Despite being recognized as a distinct clinical entity, MCTD displays heterogeneity in the age of onset, clinical trajectory and organ involvement. Paediatric-onset MCTD is uncommon and generally associated with low mortality; however, its diagnosis and management remain challenging [1, 2]. Although anti-U1-RNP antibodies serve as a diagnostic hallmark, they also occur in SLE and SSc, often obscuring early classification [3]. Pulmonary involvement, particularly interstitial lung disease (ILD), represents a key determinant of prognosis in MCTD [4]. ILD in paediatric patients is often under-recognized and warrants careful evaluation [5]. Recently, antibodies directed against the survival motor neuron (SMN) complex have been proposed as biomarkers linked to ILD and heightened disease activity [6]. However, the clinical significance of anti-SMN complex antibodies in paediatric MCTD, particularly in relation to ILD, remains unclear. Here, we report a paediatric case of anti-SMN complex antibody-positive MCTD complicated by ILD and evaluate the serological association between SMN and RNP-related antibodies using multiplex autoantibody profiling.

A 9-year-old previously healthy girl presented with painless Raynaud’s phenomenon for several months (Fig. 1A). Dermatologic assessment suggested a systemic autoimmune disease, prompting referral to our department. On admission, she showed left cervical lymphadenopathy, digital colour changes and mild sausage-like swelling of the fingers, while the nailfolds showed no capillary abnormalities (Fig. 1B and C). No respiratory symptoms were observed and chest auscultation was unremarkable. Laboratory findings revealed elevated ESR (36 mm/h), IgG (3247 mg/dl), AST (103 U/l), ALT (150 U/l), KL-6 (657.4 U/ml) and sIL-2R (2372 U/ml), with low complement levels (C3 72 mg/dl, C4 5 mg/dl). Antinuclear antibody titres exceeded 1:1280, and anti-U1-RNP antibodies were positive. No other findings suggestive of autoimmune or infectious aetiologies were identified. These findings led to a diagnosis of MCTD with organ involvement. Chest CT, performed without sedation, demonstrated bilateral dorsal ground-glass opacities in the lower lobes, consistent with ILD (Fig. 1D). Spirometry suggested a restrictive ventilatory defect with mild reduction in FEV1 (Z-score: −2.31) and FVC (Z-score: −2.44) with a normal FEV1/FVC ratio. Unfortunately, this could not be confirmed by a reduction in total lung capacity, as the patient was unable to complete the testing. Similarly, the patient was unable to perform diffusion testing. Comprehensive autoantibody screening using A-Cube (Fushimi Pharmaceutical Co., Tokushima, Japan), which quantifies 47 autoantibodies simultaneously, identified anti-SMN complex antibodies along with anti-U1-RNP_70, anti-U1-RNP_C, and anti-U1-RNP_A (Supplementary Table S1). Echocardiography revealed no cardiac complications. Prednisolone (1 mg/kg/day) improved KL-6, ESR, and imaging findings. Treatment with tacrolimus and methylprednisolone pulse therapy (30 mg/kg/day for 3 days) resulted in further improvement. The patient remains in a stable condition under follow-up (Fig. 1E and F).

**Figure 1:**
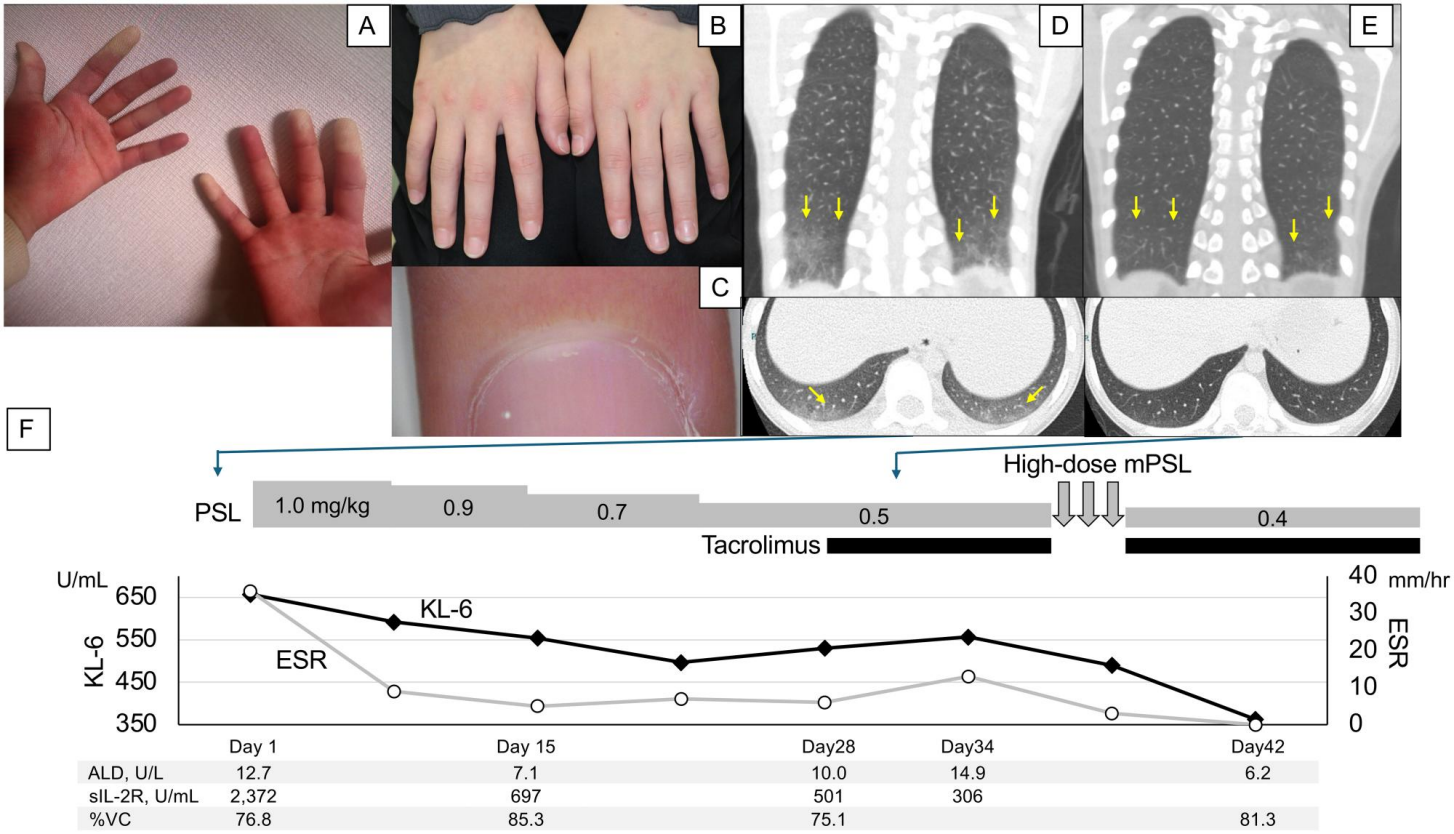
Initial findings and clinical course of the patient with paediatric MCTD. The patient exhibited Raynaud’s phenomenon (A), mild sausage-like swelling of the fingers (B) and nailfolds showing no capillary abnormalities or periungual erythema (C). (D) Chest CT revealed bilateral dorsal ground-glass opacities in the lower lobes, consistent with mild interstitial lung disease (ILD) associated with elevated serum KL-6 and ESR. (E) CT findings resolved in parallel with reductions in KL-6 and ESR levels in response to treatment. In panels D and E, coronal CT images are shown in the upper panels and axial CT images in the lower panels. (F) Clinical course showing treatment interventions and corresponding improvements in CT findings, KL-6 and ESR, together with changes in percentage of vital capacity (%VC) and aldolase (ALD). These changes reflect the treatment response and longitudinal disease activity. Abbreviations: mPSL: methylprednisolone; PSL: prednisolone

To clarify the relationship between SMN and RNP-related antibodies, sera from five paediatric MCTD patients, including this case (Supplementary Table S2), were analysed using A-Cube. Pearson’s correlation analysis demonstrated positive associations between anti-SMN and anti-U1-RNP_70 (*r* = 0.98, *P* = 0.003) and anti-U1-RNP_C (*r* = 0.95, *P* = 0.012), whereas other RNP subtypes showed weaker correlations (*r* ≤ 0.53, Supplementary Fig. S1). These findings suggest close antigenic or structural relationships between SMN and U1-RNP subunits in paediatric MCTD.

This case represents a rare paediatric presentation of MCTD complicated by ILD, in which the detection of anti-SMN complex antibodies provides new insight into their clinical relevance in childhood-onset MCTD.

ILD is a major cause of morbidity and mortality in adult MCTD, affecting 50–85% of patients [4, 7]. In paediatric cases, ILD is reported in ∼25%, with reduced diffusion capacity in one-third, while pulmonary hypertension occurs in 1.8–6.7% [1, 7]. These findings highlight the need for regular imaging and pulmonary function monitoring even in asymptomatic children [8]. Serum KL-6 is a useful biomarker of disease activity and pulmonary involvement [4]. In our patient, CT and spirometry findings with elevated KL-6 indicated subclinical yet active ILD.

Anti-SMN complex antibodies, first identified in inflammatory myopathies and overlap syndromes, are detected in up to 40% of MCTD cases and correlate with ILD, pulmonary hypertension and poor outcomes [6]. SMN is known to act as a splicing regulator within the spliceosome complex, which includes U1-RNP and other splicing cofactors [9]. Autoantigens in MCTD may therefore reflect polyclonal immune responses against spliceosome-related components, contributing to disease heterogeneity in childhood. These findings support evidence that the present patient tested positive for multiple autoantibodies against U1-RNP_70, U1-RNP_C, and anti-U1-RNP. In children, immature immunoregulation may promote early epitope diversification. The presence of ILD in our patient aligns with adult data linking anti-SMN antibodies to pulmonary involvement [6]. However, the absence of nailfold capillary abnormalities suggests comparatively mild vascular involvement in childhood-onset MCTD.

Beyond their diagnostic value, anti-SMN antibodies could serve as biomarkers for disease monitoring and stratification. Longitudinal profiling of SMN- and RNP-related antibodies, combined with molecular and clinical indices, may help identify patients at risk of progressive lung involvement or poor therapeutic response. However, a limitation of this case is that pulmonary function testing was limited by age-related technical constraints, and the small sample size warrants cautious interpretation.

In conclusion, this case highlights the potential role of anti-SMN complex antibodies as diagnostic and prognostic biomarkers in a clinically distinct subset of paediatric MCTD associated with ILD. Larger multicentre studies with longitudinal serological and clinical data are warranted to clarify their pathogenic mechanisms and therapeutic potential.

## Ethics approval

The patients and family members were studied after their informed consent was obtained. This study was certified by the Institutional Review Board of Kyushu University (no. 531–03).

## Supplementary Material

rkag073_Supplementary_Data

## Data Availability

The data underlying this article will be shared on reasonable request to the corresponding author.
