# Supplementary material for: Anti-SMN complex antibodies in paediatric mixed connective tissue disease with interstitial lung disease: clinical and immunological insights
Source: Rheumatol Adv Pract. 2026 Jul 2;10(3):rkag073. doi: 10.1093/rap/rkag073 (PMC13355598; doi:10.1093/rap/rkag073)
Supplement: rkag073_Supplementary_Data [file rkag073_supplementary_data.docx]

## Online supplementary material

**Methods**

***Participants and ethics***

Five pediatric patients including the present index case, who received the diagnosis of mixed connective tissue disease (MCTD) in Kyushu University Hospital and Fukuoka Children’s Hospital between 2012 and 2025 were enrolled in this study.

All patients fulfilled either Kasukawa’s or Alarcón-Segovia’s classification criteria for MCTD, demonstrated anti-U1-RNP antibody positivity, presented the disease before 18 years of age. Clinical characteristics, laboratory findings, and treatment histories were retrospectively collected from medical records. Serum samples obtained during active disease were used for serological testing. This study was approved by the institutional ethics committees of both institutions, and written informed consent was obtained from the patients’ guardians.

***Serological and Correlation Analysis***

To investigate the immunological relationship between anti-SMN complex antibodies and RNP-related autoantibodies, we analyzed serum samples from these five patients using A-Cube^®^ (Fushimi Pharmaceutical Co., Tokushima, Japan), a fluorescence-based multiplex immunoassay quantifying 47 autoantibodies simultaneously [1, 2]. Antibody titers against SMN, U1-RNP_70, U1-RNP_C, U1-RNP_A, total RNP, and U2-RNP were obtained according to the manufacturer’s protocol, and values >10 U were defined as positive. Values exceeding the upper detection limit were assigned the maximum measurable titer for analysis.

Pearson correlation coefficients (r) were calculated to evaluate associations between anti-SMN and RNP-related antibody titers. Ninety-five percent confidence intervals (95% CIs) were derived using Fisher’s z-transformation. All analyses and visualizations were performed in Python 3.11 using the scipy.stats and matplotlib libraries.

## Supplementary Table S1. Autoantibody profiling by A-Cube^®^ in the present case

| Antibodies | Antigens | Result |
| --- | --- | --- |
| Jo-1 | HARS | 0.1 |
| PL-7 | TARS | 0 |
| PL-12 | AARS | 0 |
| EJ | GARS | 0 |
| KS | NARS | 0 |
| OJ | IARS | 0.1 |
|  | EPRS | 0 |
|  | LARS | 0.1 |
|  | MARS | 0.2 |
|  | QARS | 0.4 |
|  | KARS | 0.2 |
|  | RARS | 0 |
|  | DARS | 0.2 |
|  | AIMP1 | 0 |
|  | AIMP2 | 0.7 |
|  | AIMP3 | 0.1 |
| Zo | FARSA | 0.5 |
|  | FARSB | 0.1 |
| Ha | YARS | 0 |
| SRP | SRP54 | 0 |
|  | SRP14 | 0.2 |
|  | SRP19 | 0.3 |
|  | SRP68 | 1.4 |
|  | SRP72 | 1.9 |
| Mi-2 | CHD3 | 0.1 |
|  | CHD4 | 0.1 |
| TIF1-γ (p155) | TRIM33 | 0 |
| TIF1-α (p140) | TRIM24 | 0.1 |
| TIF1-β | TRIM28 | 0 |
| MJ (NXP-2) | MORC3 | 0.2 |
| SAE | SAE1 | 0 |
|  | UBA2 | 0.4 |
| **SMN** | **SMN1** | **52.7** |
| cN1A | NT5C1A | 3.7 |
| **U1-RNP_70** | **SNRNP70** | **450** |
| **U1-RNP_A** | **SNRPA** | **71.4** |
| **U1-RNP_C** | **SNRPC** | **590.7** |
| **U2-RNP** | **SNRPB2** | **126.3** |
| Ku | XRCC5 | 0 |
|  | XRCC6 | 0.7 |
| PM-Scl100 | EXOSC10 | 1.8 |
| PM-Scl75 | EXOSC9 | 0 |
| RUVBL1&2 | RUVBL1&2 | 0.4 |
| Ki | PSME3 | 0.5 |
| **SS-A/Ro52** | **TRIM21** | **19.8** |
| SS-A/Ro60 | TROVE2 | 3.7 |
| SS-B | SSB | 0.4 |

Positive cutoff: >10 index according to manufacturer’s criteria (Fushimi Pharmaceutical Co., Ltd., Tokushima, Japan)

**Supplementary Table S2. Clinical and Serological Features of Pediatric Patients with MCTD in our cohort**

| Variable | Case 1 | Case 2 (Index) | Case 3 | Case 4 | Case 5 |
| --- | --- | --- | --- | --- | --- |
| Age at diagnosis, years | 8 | 9 | 9 | 11 | 13 |
| Sex | Male | Female | Female | Female | Female |
| Initial manifestations | Raynaud,  fever, rash | Raynaud | Raynaud | Facial erythema, alopecia | Easy fatigability |
| ILD by CT imaging | No | Yes | No | No | No |
| %VC, % | 78.5 | 76.8 | 101.7 | 88.9 | 88.3 |
| KL-6, U/ml | 155 | 657.4 | 158 | 213 | 269 |
| CK, U/L | 298 | 65 | 107 | 25 | 42 |
| Aldolase, U/L | 9.4 | 12.7 | 6.8 | 19 | NA |
| Nailfold abnormality | No | No | No | Yes | No |
| *Autoantibodies* |  |  |  |  |  |
| ANA, titer | 1:2560 | 1:1280 | 1:2560 | 1:2560 | 1:2560 |
| dsDNA, U/L | Negative | Negative | Negative | Negative | Negative |
| RNP, U/ml | 120.8 | >200 | 128.5 | 19 | >240 |
| Jo-1, U/ml | Negative | Negative | Negative | Negative | Negative |
| ARS, index | NA | Negative | Negative | NA | NA |
| SMN, index | 2.4 | 52.7 | 1.2 | 0.8 | 0.7 |
| U1-RNP_70, index | 75 | 450 | 68.3 | 7.9 | 99.4 |
| U1-RNP_A, index | 49.6 | 71.4 | 108.9 | 5.3 | 129.2 |
| U1-RNP_C, index | 46.9 | 590.7 | 22.6 | 2.8 | 185.7 |
| U2-RNP, index | 15 | 126.3 | 105.5 | 1.5 | 105.6 |
| Therapies | High dose-mPSL,  PSL, MTX, Tacrolimus, AZA, ABT,  RTX, Baricitinib | High dose-mPSL, PSL,  Tacrolimus | PSL | PSL, MTX, Tacrolimus | High dose-mPSL, PSL,Tacrolimus, ABT, MMF, HCQ |
| Outcome | Alive | Alive | Alive | Alive | Alive |

Abbreviations: PSL, prednisolone; mPSL, methylprednisolone; MTX, methotrexate; AZA, azathioprine; MMF, mycophenolate mofetil; RTX, rituximab; ABT, abatacept; HCQ, hydroxychloroquine; ILD, interstitial lung disease; %VC, percentage of vital capacity; CK, creatine kinase; ANA, antinuclear antibody; RNP, ribonucleoprotein; ARS, aminoacyl–tRNA synthetase; SMN, survival motor neuron


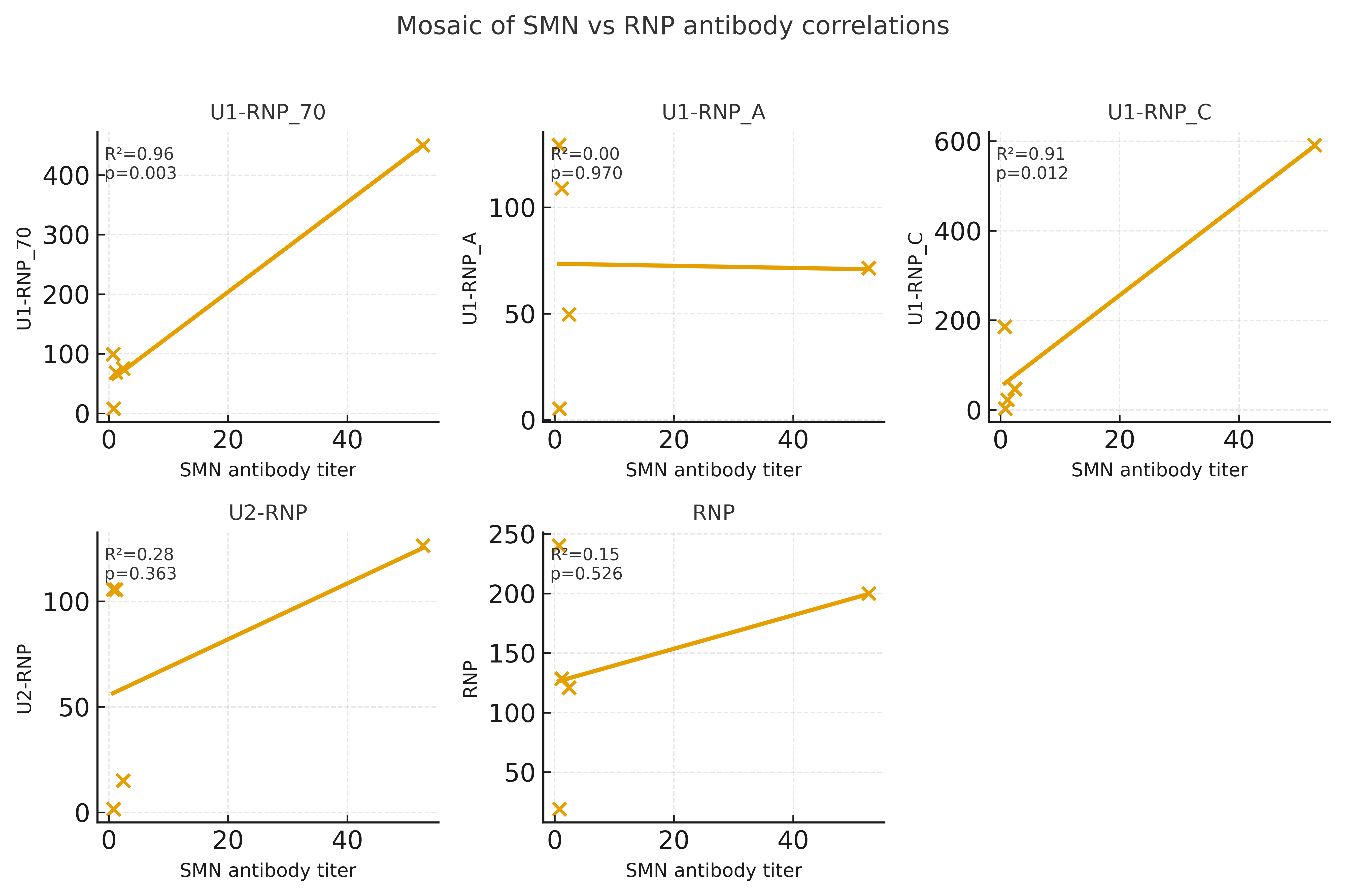
**Supplementary Figure S1. Correlation spectrum of anti-SMN antibody with each RNP-related autoantibody in pediatric MCTD**

A horizontal bar chart displays Pearson correlation coefficients (r) between anti-SMN antibodies and RNP-related autoantibodies measured in five pediatric MCTD patients. Strong correlations were observed with U1-RNP_70 and U1-RNP_C, whereas U1-RNP_A, total RNP, and U2-RNP showed weaker associations. Error bars indicate 95% confidence intervals. These findings highlight selective immunological links between anti-SMN and specific U1-RNP subunits.

**Supplementary References**

1 Norimatsu Y, Matsuda K, Yamaguchi K, et al. The Autoantibody Array Assay: A Novel Autoantibody Detection Method. DIAGNOSTICS 2023;13(18).

2 Kuzumi A, Norimatsu Y, Matsuda K, et al. Comprehensive autoantibody profiling in systemic autoimmunity by a highly-sensitive multiplex protein array. FRONTIERS IN IMMUNOLOGY 2023;14.
